# ASO Author Reflections: Induced Bias Due to Crossover Within Randomized Controlled Trials in Surgical Oncology

**DOI:** 10.1245/s10434-018-6771-4

**Published:** 2018-09-20

**Authors:** George Garas

**Affiliations:** 1 Surgical Epidemiology Unit, Department of Surgery and Cancer, Imperial College London, St. Mary’s Hospital, London, UK; 2 Department of Surgical Research and Innovation, The Royal College of Surgeons of England, London, UK

## Past

Randomized controlled trials (RCTs) provide the highest level of evidence informing clinical practice; however, their implementation in surgical oncology remains challenging.^1^ A principal reason for this is crossover from the minimally invasive surgery (MIS) arm to the open surgery arm (open conversion) due to adverse events or difficulties experienced with the MIS-tested techniques. Crossover violates the randomization process and can lead to partial homogenization of both trial arms, with survival, the primary endpoint in surgical oncology trials, being especially affected.^2^ Intention-to-treat analysis, originally developed for pharmacological trials, may not always be appropriate because, when evaluating a novel medical device or surgical technique, placebos cannot be utilized and randomization is not commonly blind.^3^ Crossover further adds to these drawbacks, and the implications can be profound. Clinical and cost effectiveness can be affected, which in turn may impact on decision making. The aims of this study were to identify modifiable factors associated with crossover and assess the impact of crossover on clinical endpoints, including mortality and complications.

## Present

The findings of this study demonstrate that crossover in surgical oncology RCTs is common, affecting one in eight patients. Moreover, its incidence was shown to reduce with increasing surgeon experience and decreasing patient comorbidity (as indicated by pretrial volume and American Society of Anesthesiologists [ASA] score, respectively). Importantly, the clinical consequences of this crossover within surgical oncology RCTs included increases in 30-day mortality, anastomotic leak rate, and complications as demonstrated by meta-regression.^4^ It thus becomes apparent that in the presence of crossover, an intention-to-treat analysis may underestimate the underlying mortality benefit associated with MIS, i.e. the benefit that would have been observed had crossover not occurred due to partial homogenization of the study groups. Similarly, the anastomotic leak rate may be overestimated in the MIS group. Hence, in the presence of crossover, any clinical, cost-effectiveness, and economic evaluation relying on traditional intention-to-treat analysis is prone to generate inaccurate results, which may impact on patient safety as well as lead to inappropriate resource allocation.^5^

## Future

Future RCTs must develop and implement strategies that include pretrial phases and surgeon credentialing by volume and/or video assessment to reduce the incidence of crossover and thus maintain randomized homogenous groups to adequately test the hypothesis. Moreover, given its independence as a predictor of crossover, as well as its potential effect on complications, it may be advisable to only include low-comorbidity patients in RCTs when initially comparing novel MIS techniques with open surgery. More complex statistical methods developed to account for the crossover effect may also need to be considered. A more pragmatic approach could involve reporting endpoints in three groups rather than two, i.e. the (completed) MIS, open, and converted (crossover) trial arms in addition to the traditional intention-to-treat analysis. This type of analysis will also allow the evaluation of factors associated with crossover, and thus try to predict which patients would not constitute good candidates for MIS due to their high risk of conversion and thus complications.
